# Stable carbon isotope diagnostics of mammalian metabolism, a high-resolution isotomics approach using amino acid carboxyl groups

**DOI:** 10.1371/journal.pone.0224297

**Published:** 2019-10-28

**Authors:** Brian Fry, James F. Carter

**Affiliations:** 1 Australian Rivers Institute, Griffith University, Nathan, Queensland, Australia; 2 Queensland Health Forensic and Scientific Services, Coopers Plains, Queensland, Australia; University of Edinburgh, UNITED KINGDOM

## Abstract

The carbon isotopic compositions of amino acids are increasingly measured to characterize diets and metabolic response to diets. We report a new high-resolution system to measure the stable carbon isotopic composition of carboxyl atoms within amino acids. The automated system used HPLC to separate amino acids followed by addition of ninhydrin for decarboxylation and transfer of the evolved CO_2_ to a stable isotope ratio mass spectrometer for *δ*^13^C_CARBOXYL_ measurement. The ninhydrin reaction was conducted at acidic pH (1.5) and elevated temperature (160 ^o^C) giving yields close to 100% for most common amino acids. Eight mammalian keratin samples from herbivores (kudu and caribou), omnivores (humans) and carnivores (bowhead and humpback zooplanktivorous whales) were analysed with this new system. The data provide an initial calibration of reference materials to be used in studies of this type and is the first report of carboxyl carbon isotope distributions in mammals. Results showed widespread ^13^C enrichments in both essential and non-essential amino acid carboxyl groups, likely linked to decarboxylation of amino acids during normal metabolism. Analyses of non-essential amino acid isotope profiles showed (1) consistent and general taxon-level metabolic differences between the herbivore, human and whale samples, (2) marked differences among individual humans, ruminants and whales (3) evidence for gluconeogenesis in the wildlife samples, and (4) extensive ^13^C enrichment likely associated with fasting in the humpback whale sample. Future mammalian research related to the metabolism of growth, reproduction, aging and disease may benefit from using this technique. Values obtained for internationally available samples USGS42 and USGS43 (Tibetan and Indian human hair) provide a first characterization of reference materials for *δ*^13^C_CARBOXYL_ profiles.

## Introduction

Carbon stable isotopes in bulk tissues such as muscle have been widely used in studies of human and animal diets and migration, tracing carbon flow from source inputs and regions. Early studies showed that in some cases, carbon isotopes are also sensitive to metabolic variation so that, for example, small (1 to 2 ‰) ^13^C enrichments were shown in the 1970s to characterize cancerous versus normal tissues [1,2]. This metabolic “fingerprint” appears more pronounced when the isotope techniques are employed at higher resolutions, e.g. in studies of specific compound classes such as lipids [3], specific molecules such as amino acids [4] or specific positions within molecules such as carboxyl positions of peptides and amino acids [5,6]. The information observed at the highest resolution, atom-specific level of isotopic variation, is calculated to equal or surpass that contained in genetics [7] and may reveal metabolic variation useful in studies of disease and aging [8].

We have recently developed the first automated position-specific isotope analysis (PSIA) system, using a well characterized ninhydrin chemistry to decarboxylate amino acids for *δ*^13^C_CARBOXYL_ isotopic analysis [9]. That system allowed laboratory tests and inter-calibrations involving pre-purified single amino acids, but was not applicable to the amino acid mixtures that are of more usual interest. These mixtures are typically obtained from protein digestions, with HPLC used to separate the hydrolysed amino acid mixtures. There were two aims of this study; first, to develop a method for the automated determination of *δ*^13^C_CARBOXYL_ values in mixtures of amino acids derived from proteins and second, to provide a first characterization of *δ*^13^C_CARBOXYL_ values in eight possible reference materials (RMs). Having obtained the first PSIA data for a suite of RMs it became possible to test an isotope model using comparative analyses of the amino acids derived from eight mammalian proteins.

The samples used in this study were all keratin proteins, including; human hair, a hoof sample from Arctic caribou, a horn sample from Ethiopian kudu and baleen of mysticete whales from both the Arctic and Antarctic. The choice of samples was determined by materials available internationally and from local sources. Although keratin has some unique properties as protein, analysing the component amino acids allowed some initial considerations of PSIA variations in mammals. We did not have whole-molecule (compound specific isotope analysis, CSIA) data for the 8 test samples, but to put the new PSIA results into perspective, we considered CSIA data from a previous study that included similar human, deer and whale samples [10]. On average, the carboxyl carbon represents one quarter of the carbon comprising an amino acid, and should be related to CSIA results that measures an average isotopic composition for non-carboxyl carbon (NCC) as well as the carboxyl carbon. In this work, we normalized *δ*^13^C results for from both PSIA and CSIA with reference to bulk *δ*^13^C values. This allowed underlying variability to be judged in an isotope metabolomics framework. As an alternative, NCC *δ*^13^C values were used in some cases to circumvent the partial self-referencing aspect that comes from using bulk *δ*^13^C measurements that contain contributions from the carboxyl carbon subcomponent.

**Fig 1:**
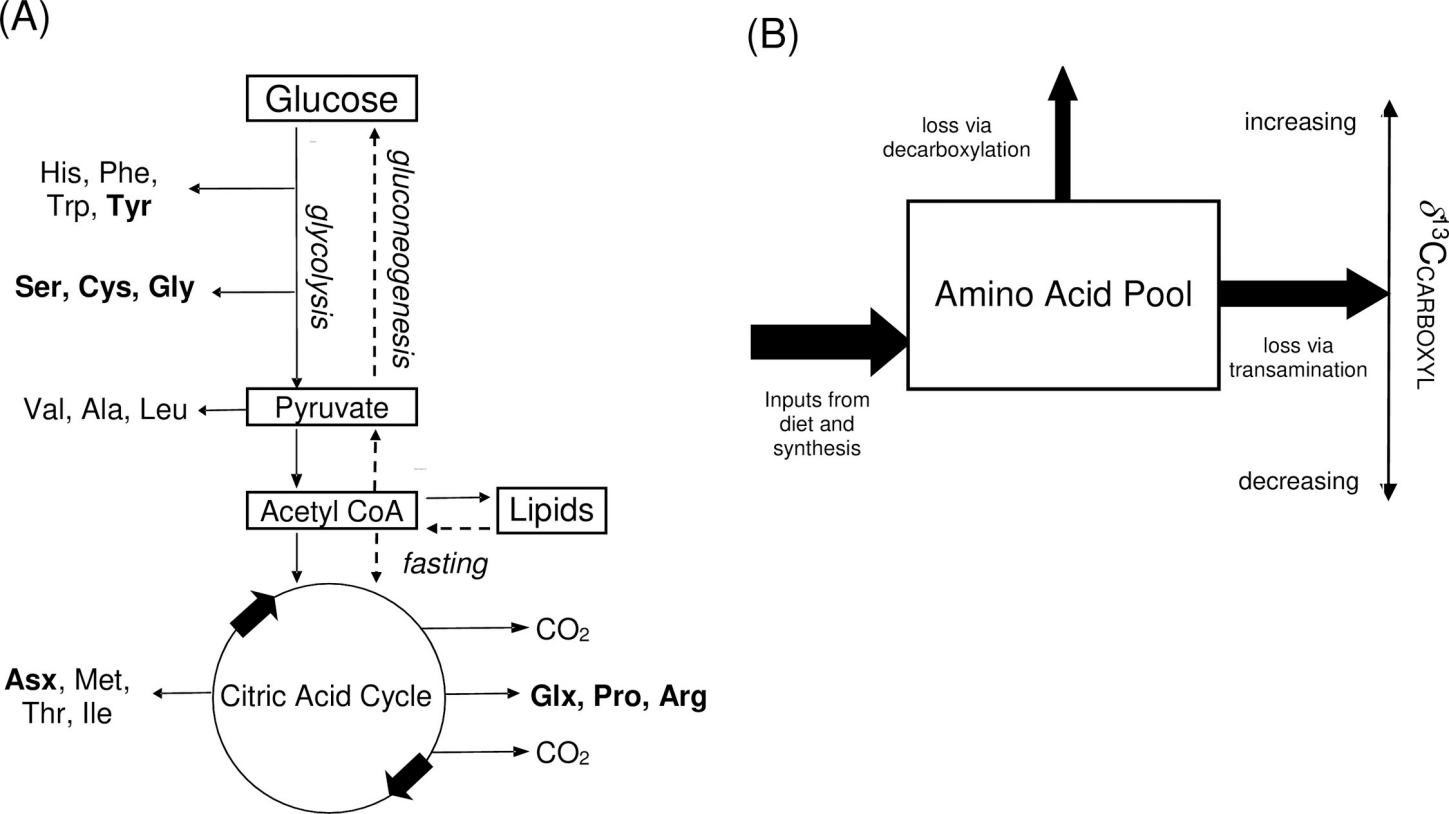
A simplified overview of amino acid metabolism linked to glycolysis, fasting and gluconeogenesis and a linked model of isotope variation in carboxyl groups of amino acids. (A) Glycolysis oxidizes glucose to CO_2_ via intermediates such as pyruvate and acetyl CoA with final oxidation in the citric acid cycle. Amino acids are synthesized from intermediates in the glycolysis network (non-essential amino acids (NEAAs) are shown in bold). During fasting, lipids are broken down for energy and can also be used to synthesize glucose in gluconeogentic pathways [11,12] (B) Observed *δ*^13^C_CARBOXYL_ can vary depending on the net balance between new inputs and losses via decarboxylation. Inputs from new synthesis and diet generally lower *δ*^13^C_CARBOXYL_ of amino acids, while fractionation during decarboxylation increases values in the residual pool. Transamination is the most common mechanism by which amino acids are degraded in mammals, but does not involve bond changes at amino acid carboxyl positions, so will have little effect on *δ*^13^C_CARBOXYL_.

The metabolism of amino acids is relatively well-characterized with intermediates of glycolytic metabolism providing the precursors for both essential amino acids (EAAs) and non-essential amino acids (NEAAs), outlined briefly in Fig 1A. Mammals acquire EAAs mostly, or exclusively, from dietary EAAs, while NEAAs are mostly biosynthesized from dietary macronutrients, with lesser contributions from dietary amino acids. In mammals, lipids are mostly biosynthesized from glycolytic precursors but these pathways can reverse. During fasting, for example, glucose is formed by gluconeogenesis and lipids are used for energy metabolism. Research has shown that reversal of normal glycolysis pathways can lead to biosynthesis of NEAAs from lipids [11] and NEAAs, such as alanine and glutamate, became rapidly labelled when fasting rats were injected with ^13^C-labelled acetate [12].

Besides effects of diet, glycolysis and gluconeogenesis, normal mammalian metabolism also involves the extensive turnover and degradation of amino acids. Most amino acid loss occurs through transamination and subsidiary losses occur by decarboxylation [13,14]. A general isotopic model for amino acid carboxyl groups (Fig 1B) shows that *δ*^13^C_CARBOXYL_ reflects the net balance between gains and losses from amino acid pools, especially recording the balance between new inputs that lead to lower *δ*^13^C_CARBOXYL_ values and decarboxylation losses that lead to higher *δ*^13^C_CARBOXYL_ values. It must be noted that isotopic differences among *δ* ^13^C_CARBOXYL_ values might be linked to varying dietary preferences and/or differences in food isotopic values as well as metabolic processes. In particular each of the NEAAs may incorporate carbon from the different dietary macronutrients in varying amounts [15,16,17]. A previous study of carboxyl isotope values in amino acids indicated that values generally decrease in food webs involving plants and invertebrates [6], but we observed widespread increases in mammalian carboxyl values that led to formulation of a new model for ^13^C enrichment (Fig 1B). The model is consistent with well-known ^13^C-enrichments during decarboxylations of intermediary metabolism [18,19] though isotope changes in most of the specific decarboxylation reactions important for the 20 amino acids remain to be studied. Thus, the model is an initial generalized construct that appeared applicable to the mammalian samples of this study, with detailed testing possible in future work. Some future exceptions and modifications might be expected to this simple general model, as for example formation of carboxyl bonds can also lead to ^13^C enrichment during fixation of bicarbonate [6], though such reactions are currently only well-described for one of the amino acids, aspartate [14].

The study of isotopic variations has been called “isotope systematics” [20], “isotopics” [21] and, when applied to biological systems, “isotopomics” [22]. Here we suggest the term, “isotomics” to connote the many large volumes (tomes) of isotope information available at the highest-resolution position-specific level.

## Materials and methods

### Samples and reference materials

Keratinaceous RMs USGS42 (Tibetan human hair) and USGS43 (Indian human hair) were purchased from the US Geological Survey Stable Isotope Laboratory (Reston, Virginia, USA). The Tibetan hair was collected from 117 individuals in a single village barbershop, while the Indian hair was commercially purchased Remy hair from 10 individuals [23]. Two further hair samples were from the authors (Carter and Fry). CHS (caribou hoof) and KHS (kudu horn) RMs were also purchased from the US Geological Survey Stable Isotope Laboratory. These samples were prepared from hooves of 8 caribou and horns from one kudu respectively [24]. Caribou and kudu are both ruminants. Two samples of baleen from filter-feeding (zooplankton-feeding) mysticete whales were also analysed. Bowhead whale baleen (BWB) was prepared from an Alaskan whale by D. Schell and used as a *δ*^2^H isotope standard [25]. Humpback whale baleen (HWB) was prepared from whale D01 as described [26,27]. Bowhead whales feed more or less continuously but undergo some fasting during migrations [28]. The humpback whale was part of an Antarctic population that feeds for only 3 months per year then fasts for the other 9 months, during migrations to and from more equatorial waters [28].

Samples of L-glutamic acid RMs USGS40 and USGS41a were purchased from the US Geological Survey Stable Isotope Laboratory. A blend of these RMs was prepared as a working RM (USGS4*) with a value of approximately +10 ‰ to assist with calibrations over the typical -30 ‰ to +10 ‰ *δ*^13^C_CARBOXYL_ range. Additional amino acid RMs were purchased from Sigma (Castle Hill, NSW, Australia) with certified purities >98%. A combined standard (15AAmix) was prepared containing alanine (Ala), arginine (Arg), aspartic acid (Asp), glutamic acid (Glu), glycine (Gly), histidine (His), isoleucine (Ile), leucine (Leu), lysine (Lys), methionine (Met), phenylalanine (Phe), proline (Pro), serine (Ser), threonine (Thr) and valine (Val). Cystine (Cys) and Tyrosine (Tyr) were not routinely included in this mixture because a significant amount of hydrochloric acid was required to solubilize these amino acids. The presence of HCl in the HPLC solvent was found to have an adverse effect on the IRMS background, especially at *m/z* 46. Because glutamine and asparagine are converted to glutamate and aspartate during acid hydrolysis (see below) combined results for glutamate plus glutamine and aspartate plus asparagine are reported as Glx and Asx respectively.

### Comparative data

For comparison with PSIA data from this study we considered CSIA results for a set of human, deer and whale samples analyzed by Choy et al in a study of paeleo-diets on the Korean peninsula [10] (S1 Table). The comparison is included primarily to give a sense of what information is available from the carboxyl carbon of amino acids via PSIA (this study) versus whole amino acid molecule via CSIA. We selected CSIA results for 8 human samples, feeding predominantly in a terrestrial food web, four deer and two whales [10]. The species of whales was not recorded but minke whales are the most common whales in that area, and are baleen zooplanktivores like bowhead and humpback whales. Although there were some differences in our study versus that of Choy et al., notably the location of samples and use of bone collagen versus keratin, both studies deal with protein amino acids formed during mammalian metabolism. Samples in both studies also represent different trophic levels in mammalian food webs, from herbivorous deer to omnivorous humans to carnivorous (zooplanktivorous) whales.

### Protein hydrolysis

Protein hydrolysis followed the method of Moore and Stein [29], with little isotopic fractionation reported for this type of acid hydrolysis [30,31]. Hair samples 10 to 30 mg were weighed into vacuum hydrolysis tubes fitted with PTFE stopcocks and 0.5 mL of 6N hydrochloric acid (Ajax Finechem, Scoresby, VIC, Australia) was added. Tubes were cooled in liquid nitrogen, evacuated and then closed. Tubes were then allowed to come to room temperature and sonicated under vacuum (“sonivac”), to remove residual dissolved oxygen, then refrozen in liquid nitrogen, evacuated and then resealed. Hydrolysis was performed by heating the closed, evacuated tubes for 20 hours at 110 ^o^C. The hydrolysates were transferred to small beakers and the hydrolysis tubes rinsed with 0.5 mL of high purity water. The combined samples were dried under high vacuum at 60 ^o^C. The dried samples were reconstituted with 0.5 mL of high purity water and filtered through a 0.22 μm nylon filters (Livingston, Rosebury, NSW Australia) into 2 mL auto-sampler vials. A second 0.5 mL aliquot of high purity water was passed through the filter to collect a total sample volume of approximately 0.8 mL.

### Instrumentation

**Fig 2:**
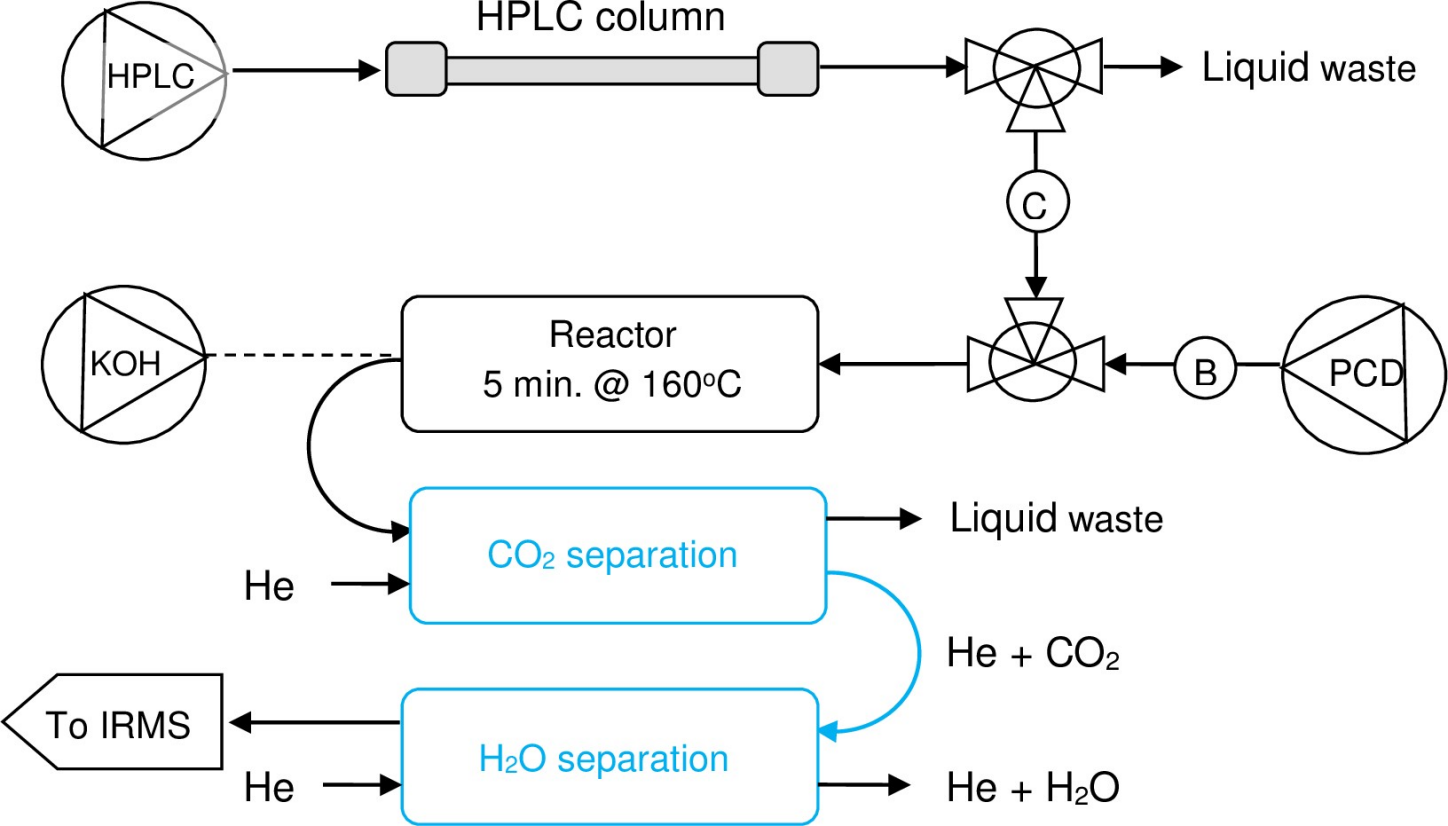
Schematic overview of the PSIA analytical instrument. Amino acids are separated by HPLC and a fraction of the eluent (150 from 750 μ l/min) is mixed 1:1 with acidic ninhydrin reagent prior to entering the 1.5 ml stainless steel coil at 160 ^o^C. Amino acids are decarboxylated as part of the ninhydrin reaction and the carboxyl CO_2_ is separated from the aqueous stream into a helium flow, dried and passed to an isotope ratio mass spectrometer to measure the carbon isotopic composition. B = back-pressure regulator, C = non-metallic check-valve. NB when the system is flushed with acetone (weekly) the connection between the CO_2_ separator and H_2_O separator must be removed.

The measurement system is shown schematically in Fig 2. Amino acids were separated using an Accela 600 HPLC pump and auto-sampler (Thermo Fisher Scientific, Waltham, MA, USA), equipped with a 4.6 mm × 250 mm × 5 μm Primesep A column and guard column (SIELC Technologies, Wheeling, IL USA). The flow from the HPLC was split using a simple T-piece with PEEK tubing restrictions to provide a flow of 0.15 to 0.16 mL min^-1^ that passed through a small-volume non-metallic check valve (Upchurch Scientific/Idex, Rohnert Park, CA, USA) to the modular PCD unit (Rigas Labs, Sindos, Greece). The majority of the HPLC eluent was directed to waste, while the flow to the PCD was mixed 1:1 (vol:vol) with ninhydrin reagent. The mixing tee in the PCD unit was replaced with a mixing tee that had a smaller (2 μ L) internal volume (VICI AG International, Schenkon, Switzerland). The PCD unit was also modified by replacing the 0.5 mL PEEK reaction coil with a stainless steel coil (0.010” i.d.) with a nominal internal volume of 1.5 mL. The stainless steel coil allowed longer reaction times at elevated temperatures with a total residence time in the reaction coil between 4.5 and 5 minutes.

The PCD reagent comprised 5.0 g spectrophotometric grade ninhydrin (ACROS, Geel, Belgium), 5.0 mL orthophosphoric acid (Ajax Finechem, Scoresby, VIC, Australia), 50 mL sulfolane (Sigma-Aldrich, St. Louis, MO, USA) and 945 mL high purity water (Milli-Q 18.2 MΩ).

The outlet from the PCD unit was connected to an LC IsoLink interface (Thermo Fisher Scientific, Bremen, Germany) that partitioned the CO_2_ into a stream of helium and dried the gas through approximately 750 mm of Nafion membrane. Overall liquid flow through the CO_2_ separation membrane in the LC IsoLink was maintained below 0.4 mL min^-1^ for optimum transfer of CO_2_ [32]. Gas from the LC IsoLink was transferred to a Delta V Advantage isotope ratio mass spectrometer (Thermo Fisher Scientific, Bremen, Germany) via two fused silica capillaries for sample (1.5 m × 100 μm id) and working gas (1.5 m × 50 μm id). Data were acquired and processed using Isodat 3.0 software.

Two elution schemes (a *short* run and a *long* run) were used sequentially to achieve optimum separation of amino acids using a three solvent system with a flow of 0.75 mL min^-1^: Both elution profiles were based on schemes developed by Smith et al. [33]. The eluents were:

A—high purity water (Milli-Q 18.2 MΩ);

B—100 mM sulphuric acid (99.999% purity from Aldrich 339741; 5.35mls/l of Milli-Q water) and

C– 10 mM potassium phosphate tribasic (Sigma, St. Louis, MO, USA) in 0.1% sulphuric acid

Because eluent C contained CO_2_ when first made, this solvent was sparged with helium for 24 hours prior to use.

**Fig 3:**
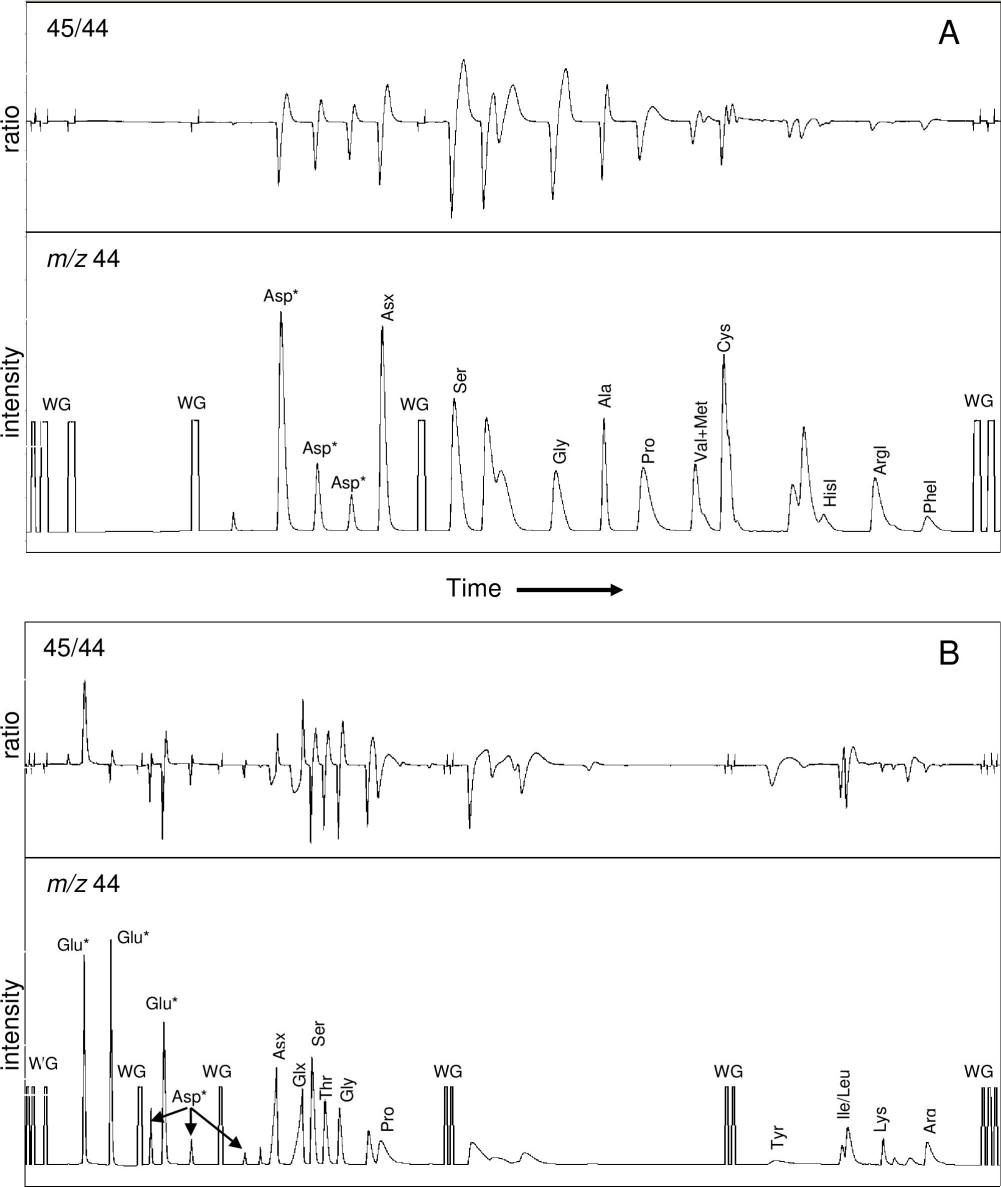
Representative ion chromatograms (*m/z* 44) and ion ratio plots (45/44) for the PSIA analysis of a keratin hydrolysate (USGS42 Tibetan human hair) showing; (A) the *short* HPLC method and (B) the *long* HPLC method. Peaks that were integrated to calculate the final results are labelled. In both chromatograms the working gas (WG) was set to an intensity of 4000 mV and the 45/44 ratio swing for the Arg peak was typically 1.16 to 1.24. The 45/44 ratio swing for the Gly peak varied between the *short* run (typically 0.9 to 1.4) and the *long* run (typically 1.10 to 1.45) due to different solvent compositions and different interaction with the ionic stationary phase.

Table 1 shows typical elution profiles for *short* and *long* runs, and Fig 3 shows typical chromatograms for USGS42 (Tibetan hair) hydrolysate. The separate *short* and *long* runs were required for baseline resolution of all the amino acid peaks, with experience showing that stable baselines for both *m/z* 44 and 45/44 ratio were necessary for reliable isotope data. The use of sequential *short* and *long* runs provided reliable results for most amino acids, with the exceptions of coeluting Val/Met and Ile/Leu. Here, we report values for the combined Val/Met peak that comprised >90% Val [34], and used a simple post-run procedure to estimate the separate values of Ile and Leu (see section 3.2, below). Peaks integrated exclusively in the *short* runs were; Ala, Arg, Cys, His, Phe and Val/Met and peaks exclusively in the *long* run were; Glx, Ile/Leu, Lys, Thr and Tyr. Other peaks (Asx, Gly, Pro and Ser) were averaged from both runs.

**Table 1:** Solvent compositions for *short* and *long* HPLC runs.

*short* run				*long* run			
time (min)	A (%)	B (%)	C (%)	time (min)	A (%)	B (%)	C (%)
				-50 pre-run (RMs)	0	0	100
				-36 pre-run (RMs)	100	0	0
-14 pre-run (RMs)	99.7	0.3	0				
0	99.7	0.3	0	0	100	0	0
				35	100	0	0
40	99.7	0.3	0				
80	0	100	0				
106	0	100	0				
				153	97.2	2.8	0
				154	32.5	67.5	0
				200	32.5	67.5	0
				201	0	100	0
				210	0	100	0

Elution profiles listed here were adjusted as the HPLC columns aged and retention properties changed. Reference materials (Glu and Asp) were introduced during the equilibration period (-50 to -14 min) prior to sample injection (0 min).

Table 2 shows a typical sequence necessary to analyse two samples in triplicate.

**Table 2:** A typical analytical sequence for two samples.

Sample run	HPLC run	comment
Asp RMs	no gradient	linearity check
Glu RMs	no gradient	calibration check
15AAmix	*short* then *long*	precision check
sample #1	*short* then *long*	replicate analysis
sample #1	*short* then *long*	
sample #1	*short* then *long*	
15AAmix	*short* then *long*	precision check
sample #2	*short* then *long*	replicate analysis
sample #2	*short* then *long*	
sample #2	*short* then *long*	
Asp RMs	no gradient	linearity check
15AAmix	*short* then *long*	precision check

During the linearity and calibration runs, Asp and Glu RMs were injected into an isocratic flow of 99.7% A / 0.3% B that had a stable baseline. Samples and the 15AAmix were analysed using a *short* run followed by a *long* run. Both *short* runs and *long* runs included RMs, injected before the sample, as additional checks of *δ*^13^C linearity and calibration; these *short* and *long* RMs eluted in regions where baselines were not entirely stable.

Analysis of the 15AAmix throughout the analytical sequence provided an estimate of the precision and accuracy of the method, with samples run in triplicate bracketed by injections of the 15AAmix (Table 2). The first run of each sequence comprised in-house Asp RMs at four concentrations to match the range in sample peak sizes and to determine whether linearity (peak size) corrections were necessary. The second run of the sequence contained several RMs including USGS40, USGS41a and USGS4*. These two runs provided long-term, between-sequence, checks on *δ*^13^C linearity and calibration and avoided interference from any small, unidentified peaks associated with protein hydrolysis.

All peak integrations were checked manually by assigning several alternate background regions, usually in front of the peak. Peaks that differed in *δ*^13^C by >2 ‰ when integrated using different background assignments were not recorded.

The bulk carbon isotopic composition of unhydrolysed keratin samples were measured by conventional continuous flow IRMS and were normalised to the VPDB scale [35]. *δ*^13^C_CARBOXYL_ values for the total hydrolysate of keratin samples (*δ*^13^C_TOTALCARBOXYL_) were measured by flow injection analysis without HPLC separation [9].

Mass balance calculations of total carboxyl carbon were made by summing the product of each measured amino acid isotopic composition and its fractional contributions to the total chromatogram.

$$\delta^{13}C_{TOTALCARBOXYL}=\sum_{i=1}^{N}\delta^{13}C_{CARBOXYL}\times fraction$$ (1)

A mass balance approach was also used to calculate non-carboxyl carbon (NCC) from the calculated bulk carboxyl and measured bulk values (Eq 2) where *f* = 0.25 i.e. the fraction of carboxyl carbon in keratins.

$$\delta^{13}C_{NCC}=\frac{\delta^{13}C_{BULK}-\delta^{13}C_{TOYALCARBPXYL}}{1-f}$$ (2)

## Results and discussion

### Notes on method development

**Fig 4:**
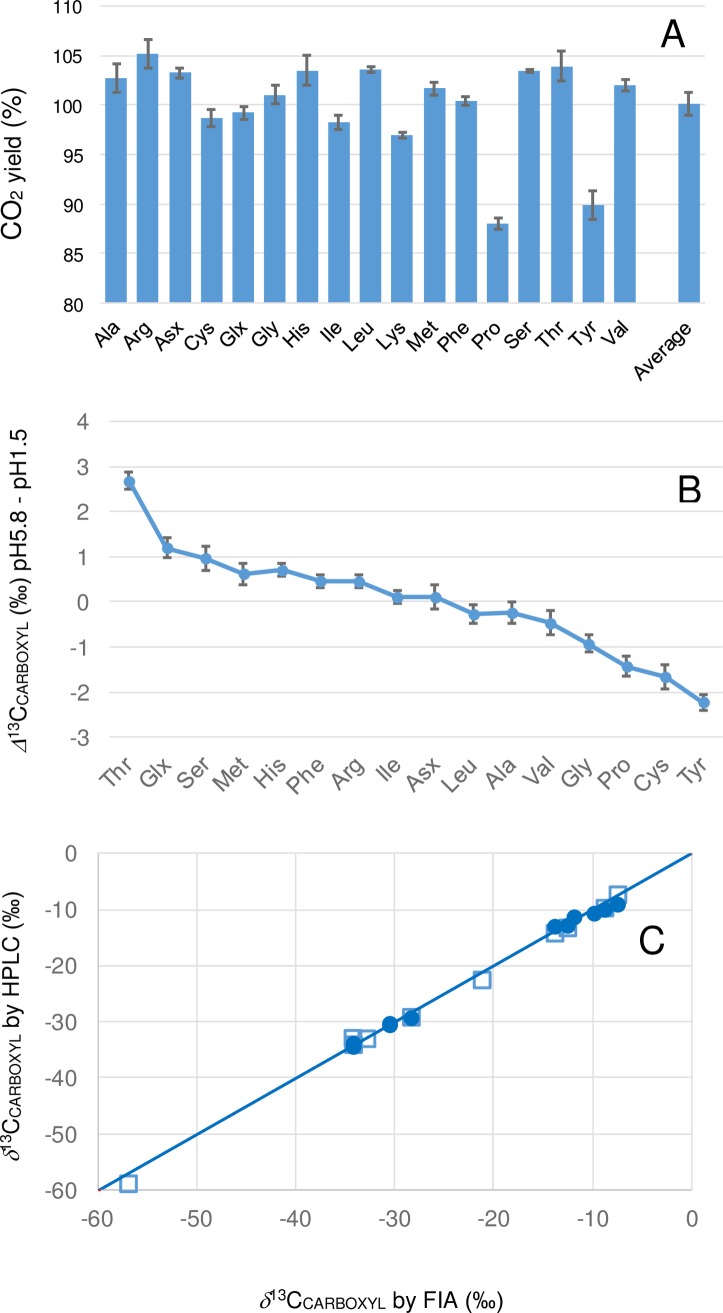
Performance considerations for the PSIA method; (A) the decarboyxlation CO_2_ yields for amino acids (mean ± sd n = 4) obtained with the pH 1.5 ninhydrin reagent, (B) the difference (*Δ*^13^C_CARBOXYL_) between amino acids decarboxyled with pH 5.8 and pH 1.5 ninhydrin reagents and, (C) a comparison of amino acid *δ* ^13^C_CARBOXYL_ values determined by flow injection analysis (no HPLC) of individual RMs and the same materials analysed by HPLC *short* (squares) and *long* (circles) runs.

We have previously reported the use of a pH 5.8 ninhyrin reagent to measure accurate and precise *δ*^13^C_CARBOXYL_ values for amino acids by flow injection analysis [9]. When combined with an HPLC eluent, however, this reagent caused three amino acids (Glu, Lys and Cys) partially to decompose and “overyield” CO_2_ with isotope values that did not solely reflect the isotopic composition of the carboxyl group. To overcome this problem we developed a ninhydrin reagent that functioned at acidic pH and did not cause Glu to decompose. We found that the acidic reagent gave CO_2_ yields that were 4 to 6% greater than obtained with the pH 5.8 reagent and not significantly different from 100% (Fig 4A). For most amino acids the *δ*^13^C_CARBOXYL_ values measured with acidic reagents showed small but consistent off-sets (typically < 1‰; Fig 4B) compared to measurements using the pH 5.8 reagent. Causes of these off-sets between the two methods remain to be resolved, but if the acidic reagent results prove to be off-set from the correct values the data in Fig 4B (S2 Table) can be used to correct the values.

The acidic chemistry described here was optimised using Gly and Lys because previous studies have shown the potential for Gly to underyield CO_2_ in acidic conditions and Lys to either underyield or overyield CO_2_ [36,37]. We generally observed that lower *δ*^13^C_CARBOXYL_ values were associated with low yields and enriched values with more complete yields. We therefore sought maximum *δ*^13^C_CARBOXYL_ values (highest δ^13^C) to optimize reaction conditions. *δ*^13^C_CARBOXYL_ values from Lys were found to maximise under reaction conditions of five minutes at 160 ^o^C. Under the same conditions *δ*^13^C_CARBOXYL_ values for Gly were slightly negative (-0.5 to -1 ‰) compared to those obtained using the pH 5.8 reagent but showed little improvement with more extreme reaction conditions, e.g. longer reaction times. Based on these results, we chose a chemistry that gave the highest *δ*^13^C_CARBOXYL_ values for Lys but slightly lower (-0.5 to -1 ‰) values for Gly. Further optimization experiments at higher temperatures were not possible with our hardware, but may be the subject of future developments, together with experiments with other decarboxylation chemistries.

Overall, we accepted the acidic chemistry as a valid way to characterize isotopic variation in amino acid *δ*^13^C_CARBOXYL_ because it avoided overyield problems (Glx, Lys and Cys) and because it gave much lower *m/z* 44 baseline values (typically 120 mV versus 600 mV) that simplified peak integration. Analysis of RMs (Table 3) showed that both methods were stable over long periods (several months) in terms of calibration and measurement of isotope differences between samples.

**Table 3:** Mean *δ*^13^C_CARBOXYL_ values for RMs run with pH 5.8 and pH 1.5 reagents.

	RM					mean	sd
	pH 5.8						
date		Mar-17	Jan-18	Mar-18			
	Val (V3) ^a^	+31.60	+31.79	+31.56		+31.68	± 0.16
	Gly (G3) ^a^	+14.70	+14.87	+14.66		+14.76	± 0.15
	Gly (WH) ^a^	-27.00	-26.82	-27.09		-26.96	± 0.19
	Ala (TTA) ^a^	-31.58	-31.28	-31.48		-31.38	± 0.14
	pH 1.5						
date			Jan-18	Mar-18	Jul-18		
	Val (V3) ^a^		+31.74	+31.75	+31.43	+31.64	± 0.18
	Gly (G3) ^a^		+14.22	+14.13	+13.77	+14.04	± 0.24
	Gly (WH) ^a^		-28.05	-27.91	-28.24	-28.07	± 0.16
	Ala (TTA) ^a^		-31.56	-31.81	-31.51	-31.63	± 0.16

All data were normalized to *δ*^13^C_CARBOXYL_ USGS40 = -30‰.

^a^ these RMs were used in the previous validation of the PSIA method [9].

A practical disadvantage of using the acidic ninhydrin reagent was precipitation of hydrindantin (a dimer of ninhydrin) in tubing leading away from the PCD unit, including the CO_2_ stripper. This tubing needed to be cleaned for five to 15 minutes daily with 0.1% potassium hydroxide solution delivered from a separate pump, shown dashed in Fig 2. Also, once a week acetone was used to flush these lines and the cooled PCD reactor. To reduce the precipitation of hydrindantin a lower concentration of ninhydrin was used in the acidic reagent (5 g/L instead of 14g/L at pH 5.8). To compensate for the reduced ninhydrin concentration the method reported here used higher reaction temperatures (160 ^o^C versus 130 ^o^C at pH 5.8). The differences in ninhydrin concentration and reaction temperature likely influenced reaction conditions [36,37] and may have contributed to the off-set isotope differences measured using the different reagents (Fig 4B).

Finally, we compared the isotope values for RMs measured by flow injection analysis or via HPLC (Fig 4C). Because deviations from the 1:1 relationship were generally small (averaging 0.5‰) we did not correct the measured data for possible chromatography-related effects. The data used to generate Fig 4C are presented in the supporting information (S3 Table) and are available if needed to correct the reported data in the future.

### Ile/Leu deconvolution

In our work, the amino acids Ile/Leu could not be separated by HPLC and so we developed a simple post-analysis procedure to estimate separate Ile and Leu *δ*^13^C_CARBOXYL_ values. The Ile/Leu peak overlap is illustrated in Fig 3, with the software simply dividing the doublet at the lowest point between the two peaks. To develop and test a deconvolution procedure a natural Leu sample (Leu1) was blended with isotopically labelled Leu (1-^13^C, 99%, Cambridge Isotope Laboratories Inc., Tewksbury, MA, USA) to give two isotopically enriched samples (Leu2 and Leu3). Each of the three Leu samples was blended with a natural L-Ile sample in a ratio similar to that found in keratin (approximately 30:70) [38,39] and Table 4 summarises the results for these experiments. The measured *δ*^13^C_CARBOXYL_ values for Leu and Ile were different in the *short* and *long* runs due to differences in separation and chromatographic fractionation. The isotopic composition of Leu made a consistent contribution to the measured isotopic composition of the Ile (and vice versa), despite an approximate 28 ‰ variation in the isotopic composition of Leu. These experiments showed that, so long as the relative concentrations of Ile:Leu remained reasonably constant, addition of empirically determined constants gave the correct isotopic compositions. For the samples of this study calculation of the Leu *δ*^13^C_CARBOXYL_ values proved robust but chromatographic interference from Tyr meant that Ile results are not reported.

**Table 4:** Illustration of the Ile/Leu deconvolution method.

	*δ*^13^C_CARBOXYL_ (‰ versus VPDB)							
	Ile				Leu			
	known	meas.	meas.-known		known	meas.	meas.-known	
Leu1	-12.21	-21.31	-9.10		-12.55	-9.66	2.89	
Leu2	-12.21	-20.91	-8.70		2.30	4.75	2.45	
Leu3	-12.21	-21.29	-9.08		15.56	18.51	2.95	
mean			-8.96	± 0.22			2.76	± 0.27

A sample of natural Ile with an assigned *δ*^13^C_CARBOXYL_ = -12.21 ‰ was blended with each of three isotopically enriched Leu samples.

### Samples

The complete analytical data for samples (S4 Table) and 15AAmix (S5 Table) analysed during this work are presented in the supporting information. The precision (sd) of *δ*^13^C_CARBOXYL_ measurements of individual amino acids derived from keratinateous RMs was typically ± 0.3 ‰ for compounds with good chromatographic separation and clean baselines in both *m/z* 44 and 45/44 traces (Fig 3) and the overall sd was 0.5 ‰ (Table 5). This precision was similar to that achieved for CSIA amino acid analysis by HPLC [10]. Analysis of three independent preparations of Tibetan hair, each analysed in triplicate, gave similar precision implying that the hydrolysis procedure did not make a significant contribution to the overall measurement uncertainty of the method. Individual amino acid *δ*^13^C_CARBOXYL_ values ranged from -36.0 ‰ for Leu in the CHS sample to -5.7 ‰ for Lys in the BWB sample. The 30 ‰ range is much wider than the 5 ‰ range predicted by thermodynamic calculations [40] but similar to that reported by previous studies [5,6]. This wide range most likely reflects kinetic, rather than equilibrium factors being dominant in intermediary metabolism including metabolism of amino acids [41]. We examined the data further after normalising to the bulk isotopic composition, a process that generally accounts for dietary differences and improves alignment between samples.

**Table 5:** *δ*^13^C_CARBOXYL_ data for amino acids derived from keratin hydrolysates.

sample	Tibetan (USGS42)^a^				Indian (USGS43)	Carter	Fry	CHS	KHS	BWB	HWB	sd ^b^
	#1	#2	#3	sd								
Ala	-19.0	-19.0	-19.4	0.3	-18.1	-19.0	-17.3	-23.2	-26.0	-22.6	-31.8	0.2
Arg	-13.3	-13.4	-13.5	0.1	-14.4	-14.1	-11.9	-12.8	-10.2	-11.7	-16.0	0.5
Asx	-16.3	-16.3	-16.5	0.1	-18.2	-17.2	-15.4	-16.2	-15.2	-12.9	-16.9	0.2
Cys	-12.4	-12.8	-13.4	0.5	-14.2	-10.6	-10.1	-15.7	-15.2	-9.7	-13.5	0.6
Glx	-14.3	-13.6	-14.0	0.4	-15.2	-14.4	-12.0	-14.1	-15.2	-13.8	-21.2	0.3
Gly	-14.2	-14.5	-14.3	0.1	-15.4	-14.2	-12.9	-14.4	-17.2	-7.1	-11.2	0.3
His	-16.2	-16.3	-13.5	1.6	-15.0	-16.2	-12.5	-17.9	-19.2	-12.8	-9.9	0.5
Ieu ^c^	-30.3	-29.3	-31.9	1.3	-34.8	-30.4	-28.2	-36.0	-33.9	-33.4	-35.3	1.2
Lys	-16.5	-16.5	-16.5	0.0	-16.8	-16.7	-14.5	-16.8	-13.6	-5.7	-7.6	0.1
Phe	-14.2	-14.8	-15.3	0.5	-16.2	-14.8	-13.5	-16.1	-17.2	-8.7	-12.9	0.6
Pro	-15.4	-15.8	-16.4	0.5	-14.4	-17.1	-14.7	-16.8	-14.9	-13.4	-16.9	0.7
Ser	-14.2	-14.4	-14.3	0.1	-15.3	-14.2	-12.8	-17.7	-17.3	-10.3	-17.0	0.3
Thr	-13.3	-14.0	-13.8	0.3	-16.4	-12.7	-10.4	-15.8	-13.7	-6.4	-7.3	0.3
Tyr	-14.1	-11.8	-12.5	1.2	-17.1	-9.3	-11.6	-20.8	-16.3	-7.7	-8.0	1.1
Val/Met	-11.5	-12.5	-12.7	0.7	-15.3	-12.5	-9.9	-15.9	-16.6	-6.2	-9.3	0.3
bulk *δ*^13^C ^c^	-21.1				-21.2	-20.4	-19.5	-22.5	-22.9	-18.7	-25.1	
total *δ*^13^C_CARBOXYL_ ^c^	-15.1				-16.2	-14.8	-13.2	-17.0	-17.0	-12.2	-16.6	
total *δ*^13^C_CARBOXYL_ ^d^	-15.8				-16.8	-15.0	-13.5	-17.8	-18.0	-12.9	-16.6	
*Δ*_CARBOXYL-BULK_	6.0				4.4	5.6	6.3	5.5	5.9	6.6	8.5	

All values are expressed in ‰ versus VPBD normalized to *δ*^13^C_CARBOXYL_ USGS40 (glutamic acid) = -30 ‰.

a data and standard deviation for three replicate hydrolysis experiments

b median standard deviation for all samples (n = 3, except for Asp, Cys, Gly, Pro, Ser n = 6)

c measured values (see text for details)

d calculated values (see text for details)

**Fig 5:**
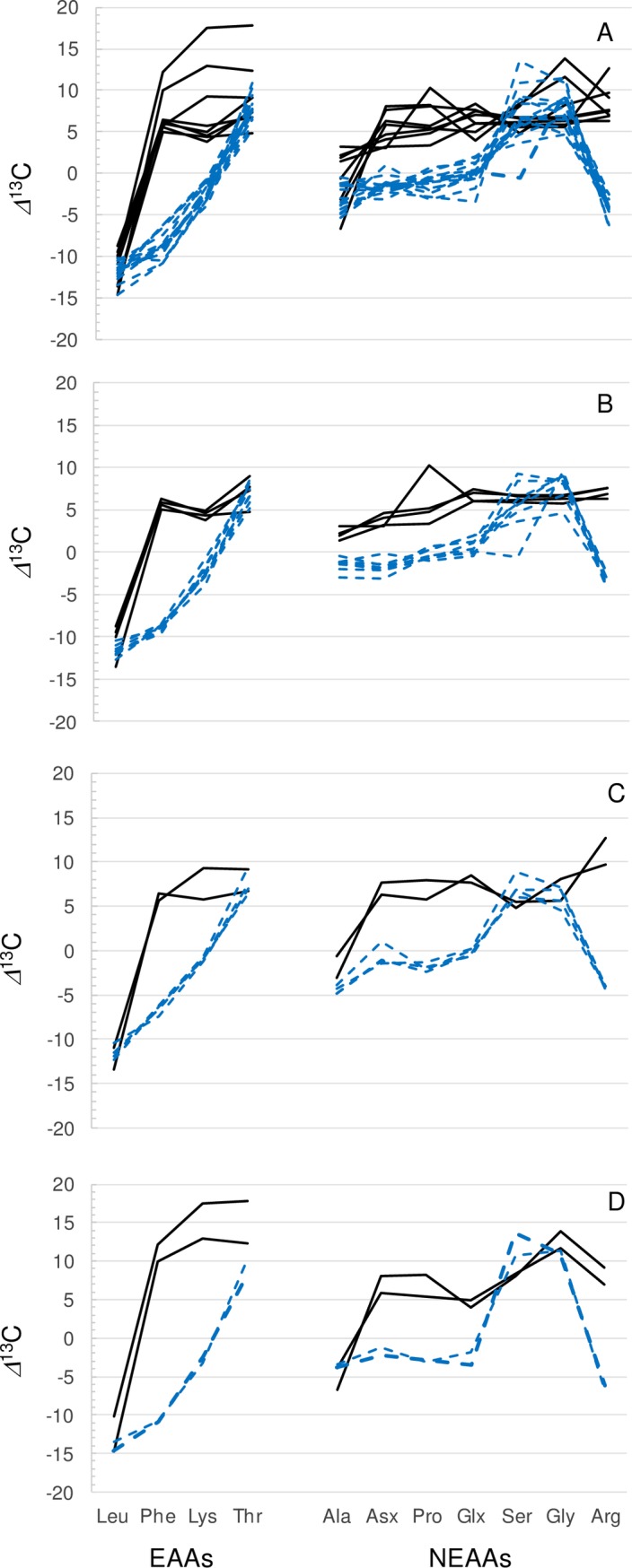
A comparison of amino acid position specific *δ*^13^C_CARBOXYL_ data from this study (solid, black lines) and compound specific *δ*^13^C data from a previous study (dashed, blue lines) [10]. Panel (A) shows data of all three taxa (humans, deer and whales), panel (B) humans, panel (C) deer and, panel (D) whales. Data are normalized to bulk carbon isotopic composition = 0‰.

The normsalised CSIA amino acid data reported by Choy et al. for humans, deer and whales spanned a similar (approximately 30 ‰) range to the normalised PSIA results for samples of this study (Fig 5). The PSIA data were, however, enriched in ^13^C for the majority (8 of 12 cases shown in Fig 5A) of the amino acids so that ^13^C-enrichment was broadly characteristic of amino acid carboxyl groups. Leu, Thr, Ser and Gly, however, had similar PSIA and CSIA *δ*^13^C values meaning that carboxyl and non-carboxyl carbon had similar isotope values. Alanine had similar PSIA and CSIA *δ*^13^C values for deer and whales (Fig 5C and 5D) but for human samples there was an average 4‰ off-set at the carboxyl position (Fig 5B). Overall, the CSIA *δ*^13^C values were very similar for different species but PSIA *δ*^13^C_CARBOXYL_ values showed many significant differences among species. This suggests that at the whole-molecule level, mammalian biochemistry operates in a uniform and consistent manner; but carboxyl groups carry a strong signal of individual metabolism. Differences in the CSIA and PSIA results showed that these data were complementary rather than correlated so that, in future studies, there is merit in both measurements.

**Fig 6:**
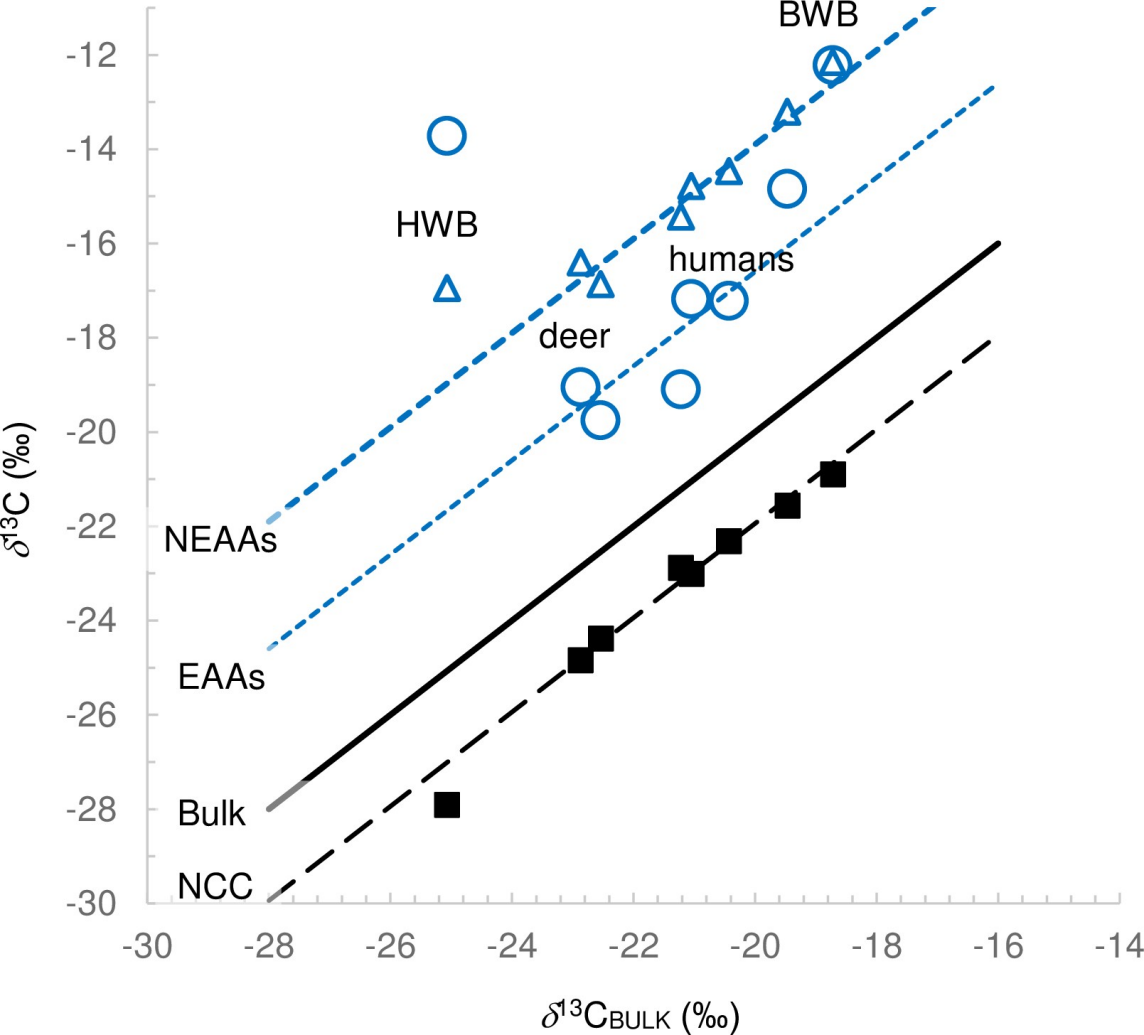
Carbon isotope compositions (concentration-weighted average) of EAA carboxyl (blue circles), NEAA carboxyl (blue triangles) and NCC (black squares). Solid black line shows bulk composition (1:1). The dashed (black) line shows an off-set of the NCC versus bulk (-1.7 ‰) and the dotted (blue) lines show an off-set of the EEAs (+2.9 ‰) and NEAAs (+6.2 ‰) versus bulk.

Possibly causes of ^13^C enrichment in the carboxyl groups were investigated. With the exception of the HWB sample, the PSIA data for NEAAs showed a strong correlation to bulk *δ*^13^C measurements (Fig 6) probably because keratin is comprised of 70% NEAAs [38]. Using Eqs (1) and (2) it was possible to estimate *δ*^13^C values of the average non-carboxyl carbon (*δ*^13^C_NCC_) in the amino acids. These were also strongly correlated with bulk *δ*^13^C values, with the exception of the HBW sample. Generally, NEAA carboxyl groups had an average off-set of 6.2 ‰ versus the bulk *δ*^13^C value and 7.9 ‰ vs. NCC.

**Fig 7:**
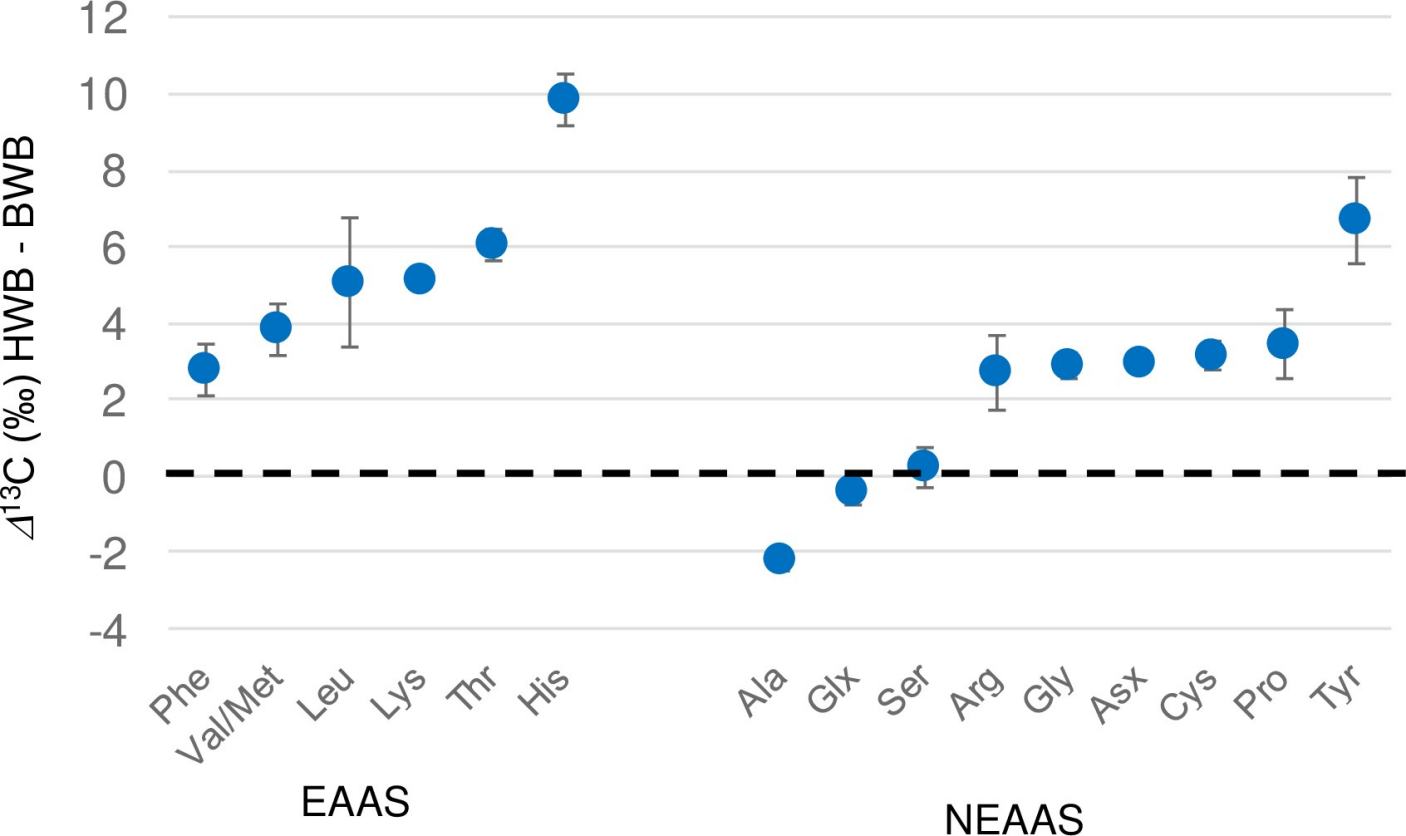
A comparison of the *δ*^13^C_CARBOXYL_ composition of individual amino acids between HWB and BWB. Data for both samples are normalised to the calculated NCC composition. Widespread ^13^C enrichment in the HWB is probably associated with the extensive fasting this species undergoes.

Compared to NEAAs, very different relationships were observed for EAAs, with deer (KHS and CHS) having the lowest ^13^C enrichments with respect to both bulk and NCC (1.6 and 3.3‰ respectively) and HBW having the greatest off-set (9.1 and 11.9 ‰ respectively). In contrast to other samples, the HBW EAAs were more enriched in ^13^C than the NEAAs (Fig 6). The ^13^C enrichment in EAAs from HWB were widespread and not confined to one or two amino acids (Fig 7). Extended fasting (> 9 months) is known for the humpback species, and widespread catabolism and decarboxylation of amino acids during fasting, accompanied by some recycling of unmetabolized amino acids, could account for these trends. EAAs are affected more by such processes because they are not renewed from the diet and represent a dwindling supply, whereas NEAAs can be continuously re-synthesized and thus represent a partially replenished stock, resulting in less overall ^13^C enrichment.

**Fig 8:**
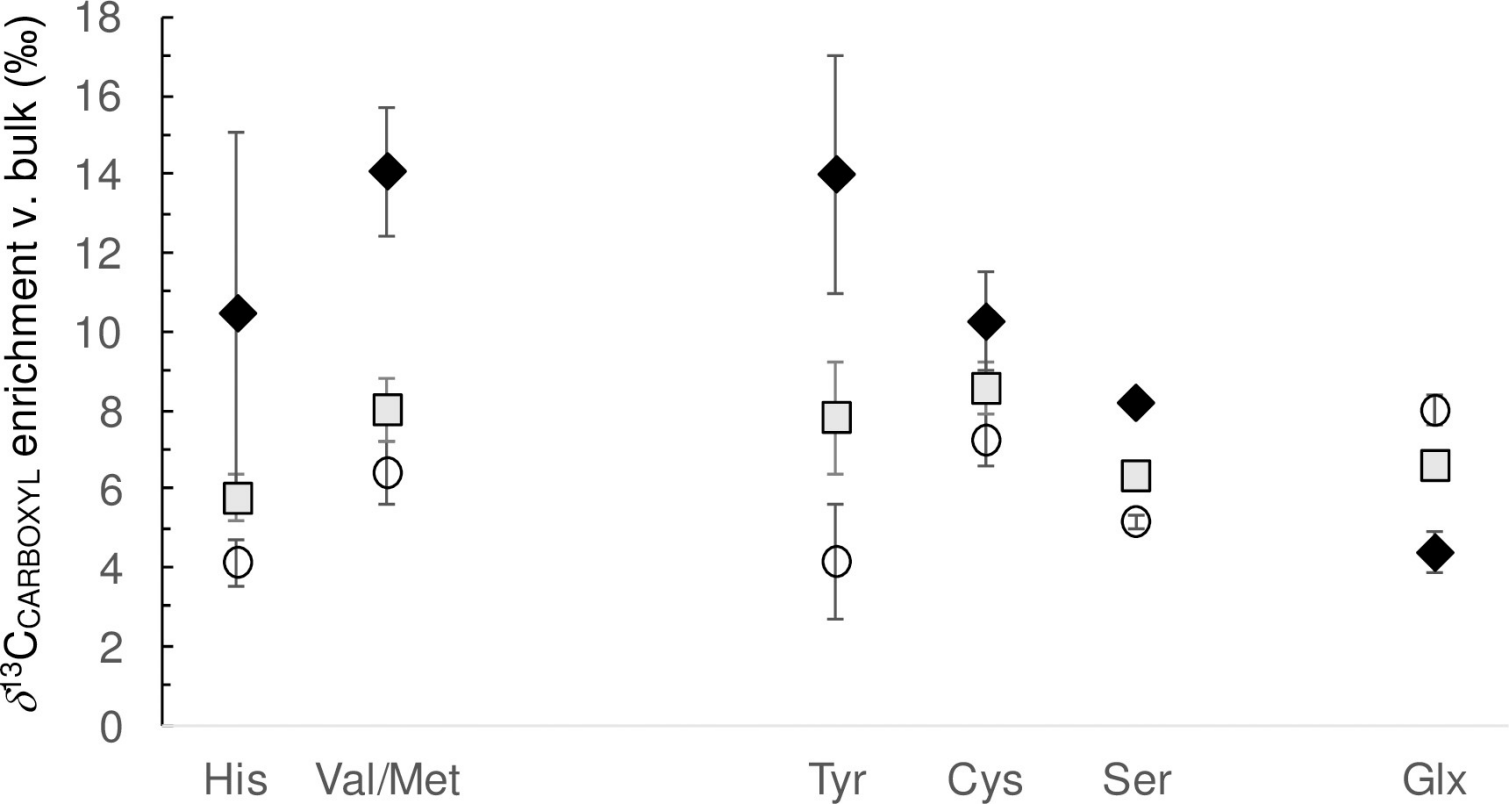
Illustration of ^13^C_CARBOXYL_ off-set (versus bulk composition) across a food web represented by herbivorous deer (open circles), omnivorous humans (grey squares) and carnivorous (zooplanktivorous) whales (black diamond), note all datum include error bars ± 1sem. For most amino acids the off-set follows a pattern whales>humans>deer but for Glx this order is reversed (deer>humans>whales) indicating ^13^C depletion across the food web for this amino acid, in agreement with trends previously reported [6].

Further insight into carboxyl ^13^C enrichment was obtained by studying trends across trophic levels, from herbivorous deer to omnivorous humans to carnivorous whales. Of the 14 amino acids studied in these mammalian samples, one (Glx; Fig 8) showed a decline in δ^13^C predicted by Savidge and Blair [6] in their studies of plants and invertebrates. Most amino acids (9 of 15) did not show a consistent increase or decrease across trophic levels, while five (His, Val+Met, Tyr, Cys and Ser) showed an increase in *δ*^13^C (Fig 8). Of these five amino acids, two were EAAs and three were NEAAs, again indicating ^13^C enrichment in both EAAs and NEAAs.

Several mechanisms for this ^13^C enrichment were considered. Decarboxylation of amino acids in catabolism or during formation of other molecules is a normal part of cellular metabolism [13], often associated with relatively large isotope effects that leave the residual pool enriched in ^13^C [18,19]. Constructing keratin from such a residual pool of amino acids would result in the observed ^13^C enrichment. Other mechanisms such as the hydrolysis of peptide bonds [42,43] or transamination appear less likely as both would result in ^15^N enrichment that has not been reported or is small in tissues such as whale keratin [27,44]. Decarboxylation of amino acids would not entail concomitant ^15^N enrichment in residual amino acids. In summary, at this time decarboxylation appears to be the simplest explanation of widespread ^13^C enrichment in carboxyl groups. Overall, the PSIA data seemed to reflect metabolic activity, with amino acid uptake from the diet and new synthesis acting to lower δ^13^C_CARBOXYL_ values while catabolic effects acting to increase δ^13^C_CARBOXYL_ values (Fig 1B). Glx was particularly interesting in this regard, with previous study indicating that Glx *δ*^13^C_CARBOXYL_ values decline in plant-invertebrate food webs to resemble those of bulk carbon [6]. For mammals, however, Glx values were greater than the bulk carbon, i.e., 4 to 8 ‰ enriched versus the bulk *δ*^13^C value. The mammalian ^13^C enrichment may represent a residual decarboxylation baseline that was not accounted for in the previous study [6].

**Fig 9:**
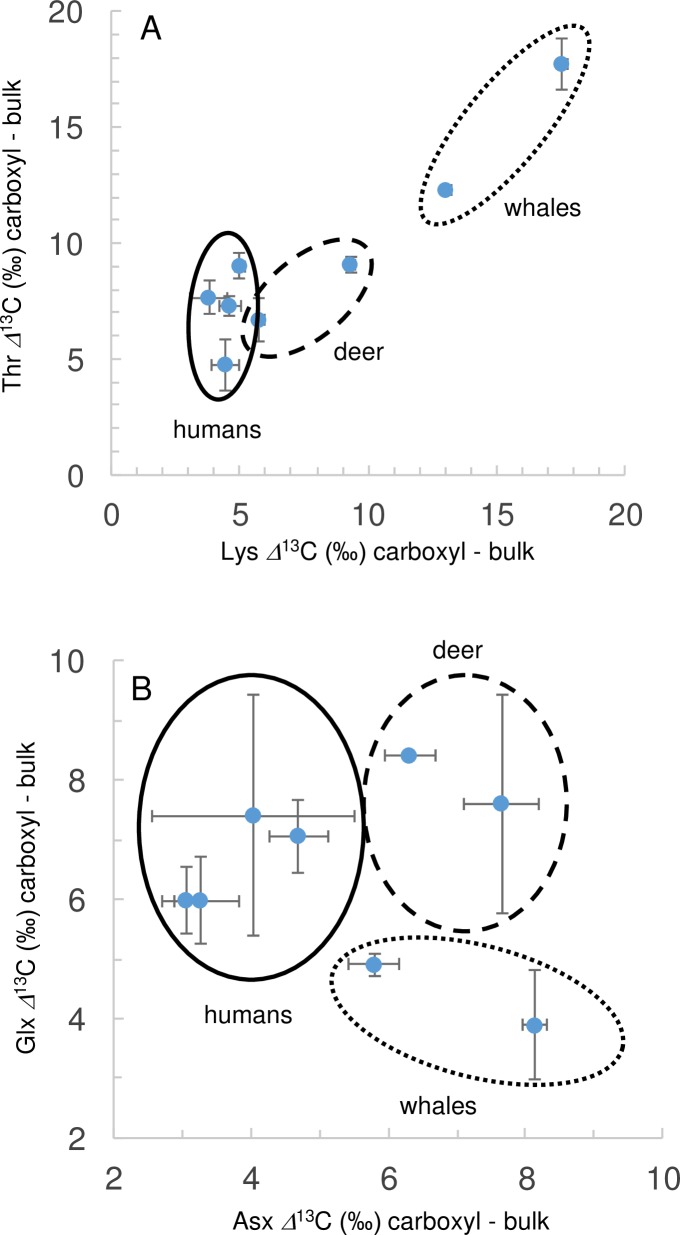
An illustration of how *δ*^13^C_CARBOXYL_ values can be used to separate the 8 mammalian samples into taxon-specific groups; humans (solid line), whales (dashed line), deer (dotted line). (A) separations based on EAAs Lys and Thr, (B) separation based on NEAAs Asx and Glx.

Such considerations suggested that the carboxyl isotope data are recording metabolic balance in a dynamic way that may lead to higher or lower carbon isotope values. A contrary null hypothesis for the most metabolically active NEAAs is that isotope signals should be well-homogenized in mammalian metabolism. This was tested by plotting the *δ*^13^C_CARBOXYL_ values of two of the most abundant and metabolically active NEAAs, Asx and Glx, along with results from two EAA indicators of diet, Lys and Thr. The EAAs separated taxa (presumably by diet), and this separation was more, rather than less, pronounced for NEAAs (Fig 9A and 9B). The NEAA data, therefore, indicated that metabolic effects were strong and persistent in the PSIA data. Similar plots using the CSIA data of Choy et al [10] showed much poorer separation, i.e. separation of only whales versus other samples with respect to Glx but not Asx. The increased degree of separation for PSIA versus CSIA data showed that the carboxyl carbon was much more sensitive to metabolic variations in these two amino acids that are central to intermediary metabolism.

**Fig 10:**
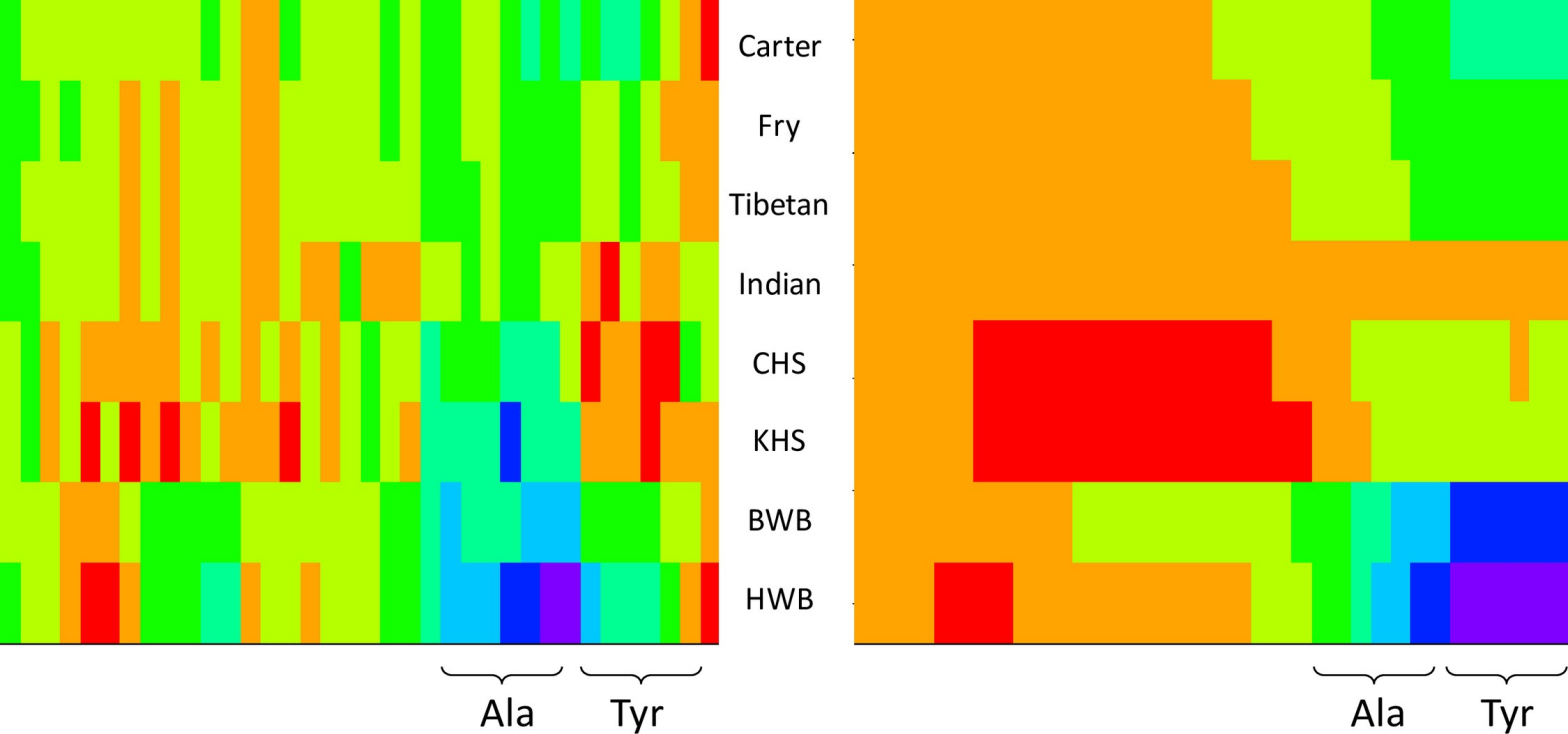
Heatmaps of *Δ*^13^C_CARBOXYL_ isotope spacings (‰) between 36 unique pairs of NEAA for the 8 study samples; (A) individual spacings and, (B) cumulative spacings. Spacings from left-to-right; Asp-Cys, Asx-Arg, Asx-Ser, Asx-Glu, Arg-Glu, Gly-Ser, Arg-Ser, Glx-Ser, Arg-Gly, Asp–Gly, Glx–Cys, Glx–Gly, Cys-Ser, Cys-Gly, Arg-Cys, Pro-Glu, Pro-Ser, Asx-Pro, Pro-Arg, Pro-Cys, Pro-Gly, Ala-Glx, Ala-Ser, Ala-Pro, Ala-Asx, Ala-Arg, Ala-Cys, Ala-Gly, Ala-Tyr, Glx-Tyr, Pro-Tyr, Asx-Tyr, Arg-Tyr, Cys-Tyr, Tyr-Gly, Tyr-Ser.

We further explored the *δ*^13^C_CARBOXYL_ data using network analysis assuming that variations in the concentrations and isotopic compositions of one amino acid can affect other amino acids. To explore these ideas, we considered differences (*Δ*^13^C) between pairs of NEAAs or isotopic spacings. For 9 NEAAs there are 36 uniquely paired *Δ*^13^C values and these differences are self-normalizing i.e. they do not need to be referenced to bulk *δ*^13^C measurements or NCC estimates. These isotopic spacings can be combined to create a heatmap profiles across the individual amino acid data (Fig 10A) or across the data considered as a cumulative average (Fig 10B) (S6 Table). It should be noted that the heatmaps in Fig 10 have arbitrary colours depending on which amino acid is first in the paired calculations, i.e., the + and–signs can reverse. If this approach is to be more widely adopted the order of amino acids in the isotope spacings will need to be standardized.

The images in Fig 10 indicate that a small fraction (10 to 15%) of the isotopic spacings had similar values across the 8 samples in this study, possibly representing a common, underlying mammalian metabolism. Samples rapidly diverged from this baseline and heatmap profiles showed the same overall groupings as outlined in Fig 9, with distinct human, deer and whale groupings, as well as characterising individuals within these groups. When data from samples with well characterised diets and metabolisms become available both multivariate and bioinformatics approaches [22] can be used to understand further this type of information-rich isotomics data.

**Fig 11:**
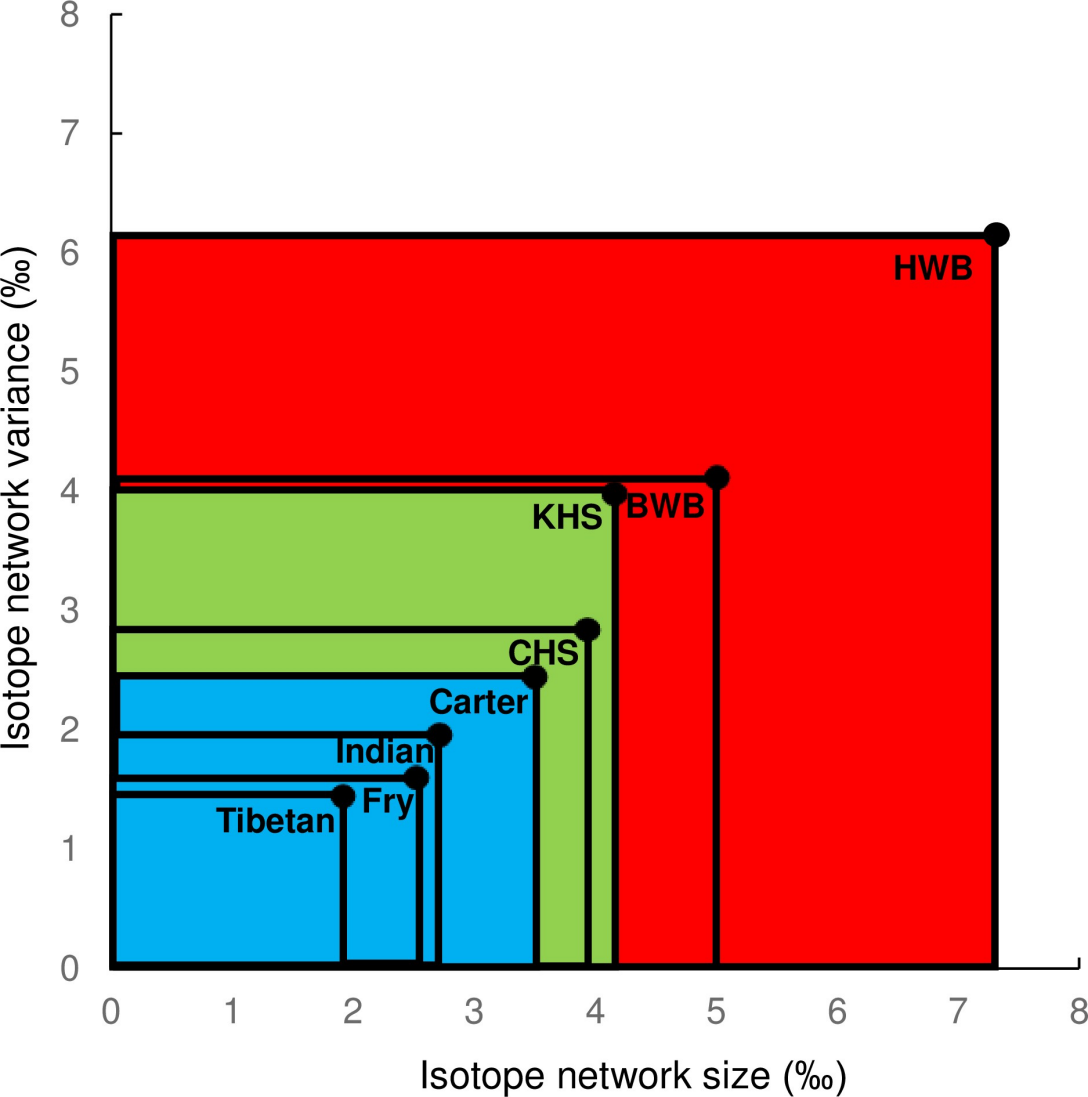
Illustration of the network size and variability for carboxyl isotope values of each sample, based on isotope spacings (average absolute values) and variability (standard deviation of absolute values).

To investigate further the networked structure of the NEAA data we calculated distance measurements as the absolute values of the isotope spacings, taking the mean and sd of these distances to represent the size and variability of the isotope metabolic networks (Fig 11). The size and variability of individual networks were positively correlated, with human samples showing small and relatively invariant networks, deer having intermediate networks, and whales the largest and most unstable (highest sd) networks. The relative uniformity and low sd of the four human networks, versus those of wildlife, may indicate a more balanced diet and regular metabolism in the humans.

Two of the NEAAs (Tyr and Ala) contributed most to network size and variability across all of the samples, with both having *δ*^13^C_CARBOXYL_ values that were divergent from other NEAAs (Fig 10). The reason for this divergence is not well known at this time but for Tyr could be related to factors such as source inputs that are not central to intermediary metabolism. Tyrosine is in low abundance in keratin [38,39] and may be less centrally linked to metabolic interconversions [14], especially because it can be formed from phenylalanine that is an essential amino acid derived from the diet. Decarboxylation of Tyr to form tyramine may also be an important control for tyrosine *δ*^13^C_CARBOXYL_ values, so that the balance between diet inputs and a specific decarboxylation pathway may lead to the large isotope variation observed for Tyr.

In contrast to Tyr, Ala is produced from pyruvate and both and both Ala and pyruvate are directly linked to glucose metabolism [14,45] (Fig 1). During normal glycolytic breakdown of glucose, Ala is expected to have high *δ*^13^C_CARBOXYL_ values because it is formed from pyruvate, with pyruvate being decarboxylated to form acetyl CoA. This decarboxylation that should leave the residual pyruvate with high *δ*^13^C_CARBOXYL_ [18,19] (Fig 1) and Ala that is formed from this enriched pyruvate should also have high *δ*^13^C_CARBOXYL_ values. When glycolytic pathways are reversed (gluconeogenesis) pyruvate is no longer decarboxylated lower *δ*^13^C_CARBOXYL_ values for pyruvate and Ala should prevail. Low isotope values of Ala during gluconeogenesis may also be due to as-yet uncharacterized isotope fractionations that occur during reactions such as decarboxylation of acetoacetate [11] or formation of propionyl-CoA [14]. Overall, the low *δ*^13^C_CARBOXYL_ data observed for Ala in the deer and whale samples appears consistent with gluconeogenesis.

It is interesting to speculate that a fasting isotope syndrome exists, with simultaneous production of isotopically ^13^C depleted Ala accompanied by a systematic and opposite ^13^C enrichment in many EAAs that are being degraded. Fasting could thus act to diverge isotope values, leading to a fasting isotope syndrome characterised by higher mean and sd of the *Δ*^13^C isotopic spacings (Fig 11). These effects may account for the large isotopic variability in the humpback whale which fasts for most of the year.

## Conclusion

This paper presents an automated method to measure the carbon isotope composition of the carboxyl groups (*δ*^13^C_CARBOXYL_) of amino acid mixtures. The components of the instrumentation were all commercially available, but were integrated for the first time in this proof-of-concept research. The limiting factor for precision and routine use of this new approach was the time and effort required to manually integrate peaks. A new generation of software using artificial intelligence could improve precision for this and other chromatography/IRMS applications. Eight samples were analysed with the new instrumentation, producing a first-ever data set for *δ*^13^C_CARBOXYL_ variations in mammals, including humans. The Tibetan and Indian hair samples (USGS42 and USGS43) may prove useful long-term RMs for PSIA work because they were typical of human hair samples and are internationally available.

The *δ*^13^C_CARBOXYL_ data for 8 samples of human, deer and whale keratin allowed detailed comparisons using 36 paired isotope spacings (*Δ*^13^C) between 9 NEAA, revealing;

consistent taxon-level metabolic differences between humans, deer and whales,
marked differences between individuals within each taxa,
evidence for gluconeogenesis in the wildlife samples,
extensive ^13^C enrichment associated with fasting in the HWB sample.

The PSIA data appeared complementary to previous CSIA studies, but provided a perspective of dietary inputs and degradation that was more “metabolic” in nature than previous carbon isotope studies. The observed increases in *δ*^13^C_CARBOXYL_ values of both EAAs and NEAAs appeared to result from decarboxylation meaning that the isotopic composition of EAAs were not just governed by inputs and mixing, but also by metabolic fractionations. The *δ*^13^C_CARBOXYL_ changes observed by PSIA measurements would often not be apparent from *δ*^13^C measurements by CSIA. For example, a 3.3 ‰ *δ*^13^C_CARBOXYL_ off-set observed for Phe versus NCC (Fig 7) would be reduced to 0.37 ‰ at the CSIA level, an off-set that could not be detected with normal 0.3 to 0.6 ‰ CSIA precision.

PSIA analyses are currently slow, about 9 to 10 hours per sample for duplicate analysis, but yield a large amount of data that can reveal how carbon is processed in intermediary metabolism. This should be a valuable addition to the field of metabolomics, adding an isotopic colour that complements studies of metabolite concentration and flux variation. It should also be possible to make a next-generation system that takes the output from HPLC chromatography and divides the flow to instruments measuring CSIA carbon and nitrogen delta-values together with PSIA *δ* values. In the longer term, different technologies will be needed to measure the non-carboxyl carbon isotope values, i.e. the remaining carbon positions in the amino acid. This study was conducted in the hope that it will stimulate technical developments for PSIA analysis, to access the big book (tome) of isotopes in this emerging field of “isotomics”, a word meant to signify the enormous amount of information available at the position-specific level of isotopic analysis.

## Supporting information

S1 Table Tabular listing of offsets between pH 5.8 and pH 1.5 chemistries, related to Fig 4B. (XLSX)

S2 Table Tabular listing of chromatography offsets between flow injection and HPLC runs, from standard runs, related to Fig 3B. (XLSX)

S3 Table Tabular listing of replicate runs for individual samples. (XLSX)

S4 Table Tabular listing of 15AAmix reference material runs associated with individual samples. (XLSX)

S5 Table Tabular listing of CSIA data from Choy et al. (2010) used for comparison with this study. (XLSX)

S6 Table Tabular listing of *Δ*spacings (‰) for paired NEAAs in the 8 study samples to support heatmap in Fig 10. (XLSX)
